# Perivascular Fat: A Novel Risk Factor for Coronary Artery Disease

**DOI:** 10.3390/diagnostics14161830

**Published:** 2024-08-22

**Authors:** Spyridon Simantiris, Aikaterini Pappa, Charalampos Papastamos, Panagiotis Korkonikitas, Charalambos Antoniades, Constantinos Tsioufis, Dimitris Tousoulis

**Affiliations:** 1 1st Cardiology Department, Hippokration Hospital, National and Kapodistrian University of Athens, 11527 Athens, Greece; spyrsim@gmail.com (S.S.);; 2 Cardiology Department, Konstantopouleio General Hospital, 14233 Nea Ionia, Greece; 3 Division of Cardiovascular Medicine, Radcliffe Department of Medicine, University of Oxford, Oxford OX1 3QT, UK

**Keywords:** perivascular adipose tissue, cardiovascular disease, atherosclerosis, pericoronary adipose tissue attenuation, fat attenuation index, coronary computed tomography angiography

## Abstract

Perivascular adipose tissue (PVAT) interacts with the vascular wall and secretes bioactive factors which regulate vascular wall physiology. Vice versa, vascular wall inflammation affects the adjacent PVAT via paracrine signals, which induce cachexia-type morphological changes in perivascular fat. These changes can be quantified in pericoronary adipose tissue (PCAT), as an increase in PCAT attenuation in coronary computed tomography angiography images. Fat attenuation index (FAI), a novel imaging biomarker, measures PCAT attenuation around coronary artery segments and is associated with coronary artery disease presence, progression, and plaque instability. Beyond its diagnostic capacity, PCAT attenuation can also ameliorate cardiac risk stratification, thus representing an innovative prognostic biomarker of cardiovascular disease (CVD). However, technical, biological, and anatomical factors are weakly related to PCAT attenuation and cause variation in its measurement. Thus, to integrate FAI, a research tool, into clinical practice, a medical device has been designed to provide FAI values standardized for these factors. In this review, we discuss the interplay of PVAT with the vascular wall, the diagnostic and prognostic value of PCAT attenuation, and its integration as a CVD risk marker in clinical practice.

## 1. Introduction

Adipose tissue, though previously underappreciated, represents now a hot topic in medical research, acting as a crucial regulator of vascular homeostasis [1,2]. Human adipose tissue is classified into white adipose tissue (WAT), brown adipose tissue (BAT), and beige adipose tissue (that presents characteristics of both BAT and WAT) [3]. WAT contributes to energy storage, thermal insulation, and mechanical protection and generates adipokines such as leptin and adiponectin that regulate metabolic homeostasis [4]. Anatomically, WAT can be classified into subcutaneous (SAT) and visceral (VAT) adipose tissue, with the latter being more strongly associated with an adverse metabolic risk profile. VAT can be divided to thoracic and abdominal VAT depots, and there are several distinct VAT subdepots with variable metabolic phenotypes. In fact, thoracic VAT includes epicardial adipose tissue (EAT), pericardial adipose tissue (PAT), and non-pericardial thoracic adipose tissue [5]. The characteristics of the aforementioned adipose tissue types are summarized in Table 1.

Perivascular adipose tissue (PVAT) around the coronary arteries belongs to EAT, but has distinct effects on local and distant physiology from the non-PVAT EAT [6]. PVAT has a unique role in cardiovascular physiology, given its anatomical proximity to the vascular wall and the absence of any anatomical barrier. It is conventionally defined as the layer of adipose tissue within a distance equal to the diameter of the artery lumen [7,8,9]. Although PVAT is categorized as WAT, PVAT adipocytes have a phenotype similar to BAT adipocytes, characterized by smaller cell size and fewer lipid droplets [10]. PVAT secretes vasoprotective adipokines under normal conditions, while dysfunctional PVAT generates pro-inflammatory and proatherogenic adipokines that affect the adjacent vascular wall. The vascular wall also affects PVAT composition, as recent evidence suggests that pericoronary adipose tissue (PCAT) around inflamed coronary arteries is characterized by increased lipolysis and decreased preadipocyte differentiation compared to normal PCAT [6,10].

A recently developed and validated biomarker called the fat attenuation index (FAI) detects these inflammation-related changes in PCAT composition as an increase in PCAT attenuation in coronary computed tomography angiography (CCTA) images [6]. FAI overcomes many of the limitations inherent to previously developed biomarkers of vascular inflammation, is highly predictive of major cardiovascular disease (CVD) events, and may track dynamic changes in coronary inflammatory burden [6,8,11]. In this review, we summarize the role of PVAT in cardiovascular pathophysiology and the clinical implications of PCAT attenuation as a novel biomarker of coronary artery disease (CAD).

## 2. Pathophysiology of EAT

EAT consists of the fat depot located between the myocardium and the visceral layer of the pericardium. EAT secretes several bioactive factors affecting the adjacent myocardium and coronary arteries, acting as a local regulator of homeostasis [12].

EAT acts as a protective tissue when the redox state balance is dysregulated in the adjacent tissues. Myocardial oxidative stress enhances antioxidant gene expression in EAT, increasing adiponectin secretion, which in turn upregulates the AMPK pathway in myocardial cells [13]. In this way, EAT mitigates nicotinamide adenine dinucleotide phosphate (NADPH) oxidase activity and limits the production of superoxide anions [14].

Vice versa, dysfunctional EAT secretes bioactive factors, such as hypoxia-inhibitory factor-1α (HIF-1α), which increase NADPH oxidase activity and thus production of reactive oxygen species (ROS) [15]. Dysfunctional EAT is also a source of pro-inflammatory adipokines, such as leptin, visfatin, omentin, interleukin-6 (IL-6), and tumor necrosis factor-α (TNF-α) [16]. ROS and pro-inflammatory factors affect the myocardium, leading to monocyte activation and myocardial fibrosis. Atrial and myocardial fibrosis may be also promoted by profibrotic molecules generated by EAT, such as matrix metalloproteinases and activin-A [17]. The excessive amount of EAT mechanically impairs diastolic function [18]. The detrimental effects of EAT dysfunction on myocardial physiology are highlighted by robust clinical data, linking epicardial adiposity to diastolic dysfunction, heart failure with preserved ejection fraction (HFpEF), and atrial fibrillation [18,19,20,21].

## 3. The Interplay between the Vascular Wall and PVAT

Though initially considered a structure supporting the adjacent vessels, PVAT is now widely accepted as an active determinant of vascular biology. In contrast to other fat depots, which influence human biology in an endocrine manner, PVAT mostly exerts paracrine effects (outside-in), affecting the adjacent vascular wall in absence of any anatomical barrier. PVAT secretes numerous bioactive factors such as adipokines, micro-ribonucleic acids (miRNAs), lipid mediators, and other molecules such as nitric oxide (NO), with either pro-inflammatory or anti-inflammatory effect [4]. Under normal circumstances, PVAT induces vasodilation through the release of adipokines such as adiponectin, omentin, apelin, and adipocyte-derived relaxing factor, which lead to opening of K^+^ channels in smooth muscle cells (SMCs), and through the release of vasodilators such as nitric oxide and hydrogen sulfide [22]. Beside their vasodilatory properties, these adipokines exert anti-inflammatory effects on the adjacent vascular wall. Adiponectin is an adipokine with well-characterized anti-inflammatory and antiatherogenic effects, as supported by preclinical and clinical studies. These effects are mediated by inhibition of nuclear factor-κB (NF-κB) signaling in endothelial cells, and thus reduction of IL-6 and TNF-α [23]. Importantly, adiponectin enhances AMP-activated protein kinase (AMPK) activity, thus promoting endothelial nitric oxide synthase (eNOS) expression in the endothelium and NO bioavailability [24]. AMPK activation is also associated with inhibition of the nucleotide-binding domain, leucine-rich–containing family, pyrin domain–containing-3 (NLRP3) inflammasome [25]. On the other hand, in patients with pro-inflammatory states like obesity or insulin resistance, AMPK seems to be downregulated due to the deleterious effects of pro-inflammatory cytokines. AMPK may constitute a novel therapeutic target to counteract the pro-inflammatory state related to insulin resistance [26]. Adiponectin secretion upregulates the expression of anti-inflammatory cytokines, such as IL-10 [27]. Similarly, omentin inhibits the production of pro-inflammatory cytokines, such as IL-6 and TNF-α, and promotes the expression of anti-inflammatory cytokines, exerting favorable effects on the endothelium and smooth muscle cells [28].

These effects are mitigated under inflammatory conditions, such as obesity, leading to a pro-inflammatory PVAT profile. In this case, PVAT generates adipokines, such as leptin, chemerin, visfatin, and resistin, which diffuse to the adjacent vascular wall, inducing vasoconstriction, SMC migration, and endothelial dysfunction [29,30]. Pro-inflammatory cytokines, such as IL-6, interferon-γ (IFN-γ), TNF-α, and monocyte chemoattractant protein-1 (MCP-1), are produced by PVAT macrophages and T-cells and contribute to local inflammation [31,32,33]. Pro-inflammatory adipokines enhance TNF-α, IL-6, and IL-12 expression in monocytes, promote oxidative stress by ROS production, and upregulate cell adhesion molecules in the endothelium. Thus, they trigger endothelial dysfunction and atherosclerotic plaque formation [34]. Recent evidence has highlighted the role of adipocytes as secretors of exosomes transferring miRNAs having either proatherosclerotic or antiatherosclerotic effects. MiR-133, miR-21, and miR-143 expression is significantly decreased in PCAT around segments with occlusive coronary plaques [35]. Expression of the pro-inflammatory miR-103-3p was higher in the PCAT of patients with CAD, while the PVAT-derived miR-382-5p abrogated the formation of foam cells [36,37].

**Figure 1:**
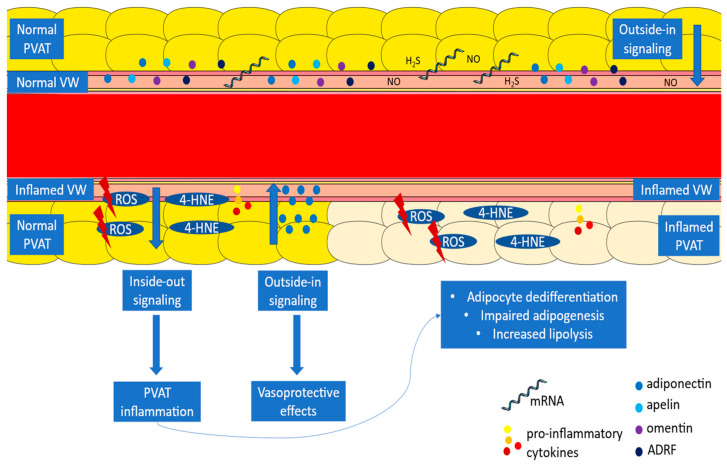
The interplay between PVAT and the vascular wall. ADRF: adipocyte-derived relaxing factor, mRNA: messenger ribonucleic acid, PVAT: perivascular adipose tissue, ROS: reactive oxygen species, VW: vascular wall, 4-HNE: 4-hydroxynonenal.

Recent evidence supports that the relationship between PVAT and the vascular wall is bidirectional, as the latter influences PVAT through paracrine signals which drive changes in PVAT secretory phenotype (inside-out) [4]. Oxidative stress in the vascular wall leads to the release of lipid peroxidation products as 4-hydroxynonenal which diffuse to the PVAT, driving an upregulation in adiponectin gene expression and adiponectin secretion. Adiponectin exerts antioxidant effects on the adjacent vascular wall [38]. In this way, PVAT acts as a protective mechanism against vascular oxidative stress. Simultaneously, pro-inflammatory cytokines, such as IL-6, TNF-α, and IFN-γ, secreted from the vascular wall or atheromatous plaques, prevent differentiation of preadipocytes to mature adipocytes and contribute to decreased lipid droplet accumulation in the PVAT [6]. Thus, a gradient of adipocyte size is created around the inflamed vascular wall, with undifferentiated preadipocytes containing low fat proximal to the vessel surrounded by larger, fat-filled adipocytes distal to the vascular wall [6]. The interplay between the vascular wall and PVAT is presented in Figure 1.

## 4. PVAT Attenuation as a Biomarker of Vascular Inflammation

The aforementioned changes in PVAT induced by vascular inflammation led to the discovery of FAI, a new imaging biomarker for detecting coronary inflammation [6,39]. Computed tomography (CT) may aid in PVAT characterization beyond volumetric measurements, as a gradient in the CT signal attenuation of PVAT [between −190 and −30 Hounsfield units (HU)] can detect inflammation-related morphological changes [6,40]. In fact, lipolysis in PVAT adipocytes and adipocyte dedifferentiation induced by pro-inflammatory cytokines lead to an increase in the water/lipid ratio in PVAT close to the inflamed vascular wall. Consequently, these morphological changes drive an increase in PVAT mean CT attenuation towards −30 Hounsfield units (HU) [6].

FAI, defined as the mean attenuation of adipose tissue within a region of interest measured from reconstructed CT images, was developed to quantify attenuation gradients in PVAT [6]. In a proof-of-principle study, FAI was firstly assessed ex vivo in explants from several fat depots (epicardial, thoracic, and subcutaneous) and found to be inversely associated with the extent of adipocyte differentiation and adipocyte size. The ex vivo findings were validated in vivo by measuring FAI from CT images of the same patients [6]. The FAI of subcutaneous adipose tissue was also positively associated with ^18^F-FDG uptake in vivo, indicating that FAI can reliably measure fat tissue inflammation, at least in certain fat depots [6]. In a subsequent study of coronary lesions in stable patients, increased PCAT attenuation was observed around coronary plaques with ^18^F-NaF uptake, further supporting the role of FAI as a vascular inflammation biomarker [41]. The value of FAI has been also assessed through radiotranscriptomic analysis of adipose tissue. Wavelet-transformed mean attenuation, a radiomic marker similar to FAI, was the best metric to identify adipose tissue inflammation, as assessed by TNFA expression [42].

Expression of genes related to preadipocyte maturation and lipid accumulation was downregulated in PVAT close (1 mm) to the right coronary artery (RCA) wall. Thus, pericoronary adipose tissue (PCAT), similar to PVAT around other arterial trees, is regulated by paracrine signals released from the human coronary artery wall [6]. To assess the ability of FAI to track coronary inflammation, FAI was measured around the proximal segment in three-dimensional cylindrical layers of 1 mm thickness, moving up to 20 mm from the adjacent vascular wall. Indeed, FAI increased progressively in layers closer to the vascular wall, where adipocytes are small and less differentiated and contain a higher water/lipid ratio.

FAI was originally measured around the proximal RCA (10–50 mm from the RCA origin), mostly due to technical factors [6]. Later studies have provided appropriate algorithms to quantify FAI around the proximal segments of the left anterior descending (LAD) and the left circumflex (LCx) [9]. The FAI around left main coronary artery has not been measured routinely because of its variable length [8].

## 5. FAI in Assessing Coronary Atherosclerosis

Taking into consideration the ability of FAI to detect coronary inflammation, its ability to track coronary atherosclerosis was assessed [6]. Indeed, pericoronary FAI (defined as the average PCAT attenuation within a distance from the coronary artery wall equal to the vessel diameter) measured around the proximal RCA was significantly lower in healthy individuals compared to patients with coronary artery disease. Pericoronary FAI was also related with atherosclerotic plaque burden in all major epicardial coronary arteries. These findings were independent of traditional CVD risk factors, age, gender, and coronary calcium score (CCS) [6]. It should be noted that CCS was not related with atherosclerotic plaque burden, indicating a possible superiority of pericoronary FAI over CCS in detecting atherosclerosis [6]. This may be partly explained by the ability of FAI to detect vascular inflammation around both calcified and non-calcified plaques, whereas CCS detects only calcified plaques. Intriguingly, pericoronary FAI varied between healthy individuals, flagging coronary inflammation even in patients with no overt coronary atherosclerosis [6].

The above findings have been also confirmed in later studies. Mean PCAT attenuation around healthy coronary segments was found to be lower compared to that around coronary plaques, either fibrous or lipid-rich (−34 ± 14 HU vs. −56 ± 16 HU). PCAT attenuation did not differ between fibrous and lipid-rich plaques, indicating similar inflammatory burden in both plaque morphologies [43].

FAI has been also associated with the hemodynamic significance of coronary lesions in several studies. In a cohort of patients with stable angina who underwent CCTA and fractional flow reserve (FFR) measurement within 2 weeks, pericoronary FAI was significantly higher around plaques with hemodynamic significance, defined as plaques with FFR ≤ 0.8 (−64 vs. −76 HU) [44]. FAI, though a modest predictor of significant coronary stenoses when used alone [area under the curve (AUC) = 0.63], was shown to be a valuable diagnostic tool for critical stenoses in combination with total plaque volume and diameter stenosis (AUC = 0.82), yielding a diagnostic performance similar to FFR-CT [44]. In line with these results, pericoronary FAI was associated with FFR of LAD intermediate stenoses in a cohort of 187 stable patients, independently predicting lesions with FFR < 0.75 (OR = 3) but not with FFR ≤ 0.8. Higher FAI values were observed around highly stenotic plaques, and pericoronary FAI enhanced discrimination of plaques with FFR < 0.75 [45]. Consequently, FAI may sense the severely increased inflammatory burden in the PVAT around hemodynamically significant stenoses. However, it does not represent a specific marker of stenosis severity, and hence it cannot replace established biomarkers of critical stenoses such as FFR [44,45].

PCAT attenuation has been also associated with myocardial perfusion assessed by PET-derived coronary flow reserve (CFR) [46]. Higher PCAT attenuation was observed around coronary arteries with impaired CFR, independent of traditional risk factors, stenosis severity, extent of coronary atherosclerosis, and high-risk plaque (HRP) features [46]. Interestingly, the association between PCAT attenuation and CFR status was significant in coronary arteries without obstructive CAD but not in coronaries with obstructive CAD. Thus, PCAT attenuation may detect impairment of vasodilation induced by coronary wall inflammation in patients without established obstructive CAD [46].

## 6. FAI as a Biomarker of Dynamic Changes in Coronary Inflammation

### 6.1. FAI as a Biomarker of Plaque Instability

Pericoronary FAI has been assessed as a plaque instability marker in several studies. In fact, it was found to be higher around culprit plaques compared to FAI around a segment proximal to the lesion in patients with recent (<3 days) acute myocardial infarction (MI) [6]. The increase in FAI around culprit lesions was independent of stent implantation in the ruptured lesion, and FAI had excellent ability to discriminate between culprit and non-culprit plaques (AUC = 0.91) [6]. Interestingly, pericoronary FAI measured around ruptured plaques in a serial CCTA scan was significantly decreased five weeks after acute MI, while no significant change was observed around stable lesions [6]. As a result, FAI represents a dynamic biomarker that is able to detect coronary inflammation related to acute plaque rupture, as well as the decrease in inflammatory burden after the acute event, acting as a “thermometer” of vascular inflammation.

Further studies also confirmed the ability of FAI to track ruptured plaques. In the studies of Goeller et al. and Lin et al., higher PCAT attenuation was measured around culprit plaques compared to around stable lesions in the same patients or highest-grade stenoses in a control group [47,48]. In another study, however, no difference was noted in PCAT attenuation between culprit and non-culprit lesions. It should be noted that the measurements of PCAT attenuation in this study were conducted in non-contrast-enhanced cardiac CT scans, thus making these findings difficult to compare with the findings from the aforementioned studies [49]. Finally, in a meta-analysis of studies comparing FAI around stable plaques to FAI around unstable plaques, FAI was indeed significantly higher around unstable plaques [50].

PCAT attenuation has been also assessed as a prognostic marker of plaque rupture events in stable patients. Higher PCAT attenuation was observed around culprit plaques for ACS compared to either non-culprit plaques from ACS patients or plaques from patients with stable CAD. PCAT attenuation did not differ between non-culprit plaques from ACS patients and plaques from stable CAD patients [51,52].

### 6.2. The Effect of Anti-Inflammatory Treatment on FAI

Pericoronary FAI has been also assessed as a sensor of changes in coronary inflammation induced by anti-inflammatory treatment. Goeller et al. observed a decrease in PCAT attenuation after statin initiation in stable patients who underwent serial CCTA imaging [53]. Similarly, another study observed a significant decrease in FAI after statin initiation in statin-naïve patients, while minimal lumen area and diameter stenosis remained unchanged. After subgroup analysis, however, this decrease remained significant only for non-calcified plaques (NCPs) and mixed plaques (MPs), but not for calcified plaques (CPs) [54]. This finding is consistent with the study of Goeller et al. correlating changes in PCAT attenuation with changes in NCP burden, but not in CP burden, which may possibly be linked to the inflammatory nature of NCPs [53,55]. Previous studies also support that the anti-inflammatory action of statins mostly affects non-calcified plaques [56]. Consequently, statins may exert an anti-inflammatory effect on coronary atherosclerosis, which can be detected by pericoronary FAI, even before these changes can be identified by CCTA as a decrease in stenosis severity.

Pericoronary FAI also significantly decreased one year after biologic therapy (anti-TNFα, anti-IL-12/23, or anti-IL-17) initiation in patients with moderate to severe psoriasis. In contrast, patients who received topical or light therapy had no significant changes in pericoronary FAI [57]. Similarly, administration of orticumab, a monoclonal antibody against oxidized low-density lipoprotein, decreased FAI in patients with psoriasis compared to placebo [58]. Thus, FAI seems to be a valuable tool in identifying changes in coronary inflammation after anti-inflammatory therapy. Future prospective studies with larger sample size and longer follow-up period are needed to explore the effect of several treatments on FAI, as also the clinical benefit of FAI reduction.

## 7. FAI as a Prognostic Tool

The Cardiovascular RISk Prediction using Computed Tomography (CRISP-CT) study was the first to explore the prognostic ability of pericoronary FAI. A post hoc analysis of prospective data from two independent cohorts of about 4000 patients who underwent clinically indicated CCTA was performed [8]. In both cohorts, pericoronary FAI values around the proximal RCA and LAD, but not around the LCx, were predictive of all-cause and cardiac mortality. Pericoronary FAI values around the RCA and LAD correlated strongly with each other, and thus most FAI measurements in the study were conducted around the proximal RCA [8]. A pericoronary FAI value of −70.1 HU was determined as the ideal cutoff in the derivation cohort, above which mortality (either cardiac or all-cause) abruptly increased (9-fold and 2.5-fold, respectively). The cutoff was also confirmed in the validation cohort. These findings were independent of gender, age, common risk factors, tube voltage, modified Duke CAD index, CCS, and presence of HRP features [8]. Moreover, FAI enhanced both all-cause and cardiac mortality risk discrimination and stratification. Adding FAI on top of a model including age, sex, CVD risk factors, CAD extent, and number of HRP features yielded significant incremental prognostic value [8]. FAI retained its incremental prognostic value even after inclusion of CCS in the prognostic model for both endpoints. However, it lost its predictive value for cardiac mortality in patients who started statin or aspirin treatment after CCTA, while it remained predictive in patients who received the same treatment after CCTA. Probably this indicates that the CVD risk detected by FAI may be modified with optimal medical therapy [8]. After subgroup analyses, FAI remained positively associated with cardiac and all-cause mortality endpoints in both primary and secondary prevention. FAI values above the cutoff were also associated with a five-fold risk of acute MI, thus linking coronary inflammation with plaque instability and acute events prospectively [8].

Consequently, CRISP-CT suggests that pericoronary FAI predicts all-cause and cardiac mortality above traditional CVD risk factors, CCS, and CCTA-derived anatomical information and may be useful in risk discrimination and reclassification in both primary and secondary prevention, flagging residual inflammatory risk. The role of CCTA in CVD prevention has been already highlighted by the Scottish Computed Tomography of the Heart (SCOT-HEART) trial, in which CCTA imaging in patients with stable chest pain was associated with initiation of more preventive measures [59,60]. This may have contributed to the significantly decreased risk of death from CAD or nonfatal MI in patients with stable chest pain referred for CCTA compared to those who received standard care [61]. In contrast to anatomical assessment of the coronary tree, FAI provides information on vascular inflammation, representing a novel imaging biomarker that can identify vulnerable patients at increased risk of cardiac mortality, even before the atheromatous plaque is formed [62]. Identification of vulnerable patients through FAI could also drive the initiation or intensification of preventive cardioprotective treatment, as evidence from the CRISP-CT study suggests that the risk identified by FAI might be modifiable by treatments such as statin or aspirin [8].

Later studies have also explored the predictive ability of FAI for cardiovascular events. Similarly, PCAT attenuation around the RCA was predictive of a composite outcome consisting of death and nonfatal MI in patients with suspected CAD, even after adjustment for clinical risk factors, CCS, HRP features, plaque volumes, and ischemia. In contrast with the CRISP-CT study, PCAT attenuation around the LAD was not related to the outcome [63]. In a cohort of type 2 diabetes (T2D) patients, PCAT attenuation around the LAD was associated with cardiovascular events after adjustment for significant stenoses and HRP features, while the association between PCAT attenuation around the RCA and outcomes was non-significant. LAD PCAT attenuation aided in CVD risk discrimination when added to a model including adverse CCTA findings (ΔAUC = 0.05) [64]. T2D has been associated with PVAT inflammation in both experimental and clinical models and is highly linked to CAD presence [65,66]. This may be attributed to an imbalance between pro-inflammatory and anti-inflammatory adipokines related to T2D, as well as to oxidative factors prevalent in insulin resistance and obesity [10,67]. Thus, FAI measurement may contribute to better CVD risk prediction and identification of vulnerable patients with T2D. FAI measurements also enhance risk stratification in patients with HRP features. A post hoc analysis of the CRISP-CT study indicated a significant amelioration in risk stratification after adding FAI status (low vs. high FAI values) on top of HRP features. Notably, HRP feature presence was not associated with an increase in CVD risk in patients with low FAI values, but patients with HRP features and high FAI were at high CVD risk (~seven-fold compared to patients with low FAI/no HRP features) [7]. FAI also independently predicted in-stent restenosis risk and severity (AUC = 0.849) after stent implantation [68].

Bengs et al. reported a significant association of FAI around RCA with major adverse cardiovascular events (MACEs) after adjustment for CVD risk factors, CCTA, and single-photon emission computed tomography myocardial perfusion imaging (SPECT-MPI) findings. However, this association was lost after adjustment for CCS, in contrast to the CRISP-CT study findings [69]. The discrepancy in the results of the subsequent CRISP-CT studies may be attributed to the use of unadjusted PCAT attenuation values and not of the standardized FAI metric, as well as to the small sample size and limited number of cardiovascular events [70]. Meta-analyses of the current literature on the prognostic value of PCAT attenuation also indicate a significant association between PCAT attenuation and MACEs [50,71].

### FAI vs. Circulating Biomarkers of Vascular Inflammation

Taking into consideration the prognostic value of pericoronary FAI, a meta-analysis compared its incremental prognostic value to that of vascular inflammation biomarkers on top of CVD risk factors [72]. Indeed, most biomarkers of vascular inflammation offered a significant increase in prognostic information over clinical risk factors. Circulating biomarkers such as C-reactive protein and IL-6 modestly improved CVD risk prognostication for the composite outcome of MACE and all-cause mortality, reflecting their low specificity for vascular inflammation [72,73]. CCTA-derived biomarkers (HRP features and PCAT attenuation) offered the highest incremental prognostic value for the composite outcome. These findings were independent of study size, follow-up length, and performance of the baseline model [72]. Consequently, CCTA imaging of PVAT for coronary inflammation quantification represents now the most valuable inflammatory biomarker to enhance CVD prognostication and could guide patient selection for anti-inflammatory treatment, especially in secondary prevention [74].

## 8. FAI in Clinical Practice

Uncorrected FAI has been validated as a useful research tool for estimation of coronary inflammation and CVD risk prognostication [8]. However, conducting FAI measurements for coronary inflammation quantification in the clinical setting in order to determine patient management is demanding. The clinical interpretation of pericoronary FAI mapping necessitates corrections for technical, anatomical, and biological factors, including age and sex. In fact, as PCAT attenuation is measured in HU, its measurement varies depending on several computed tomographic parameters. Tube voltage, reconstruction algorithm, and software, as well as CT scanner type, may account for significant variances in PCAT attenuation values [75]. Body habitus may also affect PCAT attenuation, as obese individuals have hypertrophic adipocytes, and thus lower PCAT attenuation values. In this case, PCAT attenuation may underestimate the presence of vascular inflammation [9]. Further challenges to PCAT attenuation measurement may be posed by poor image quality, coronary artery variants, and small coronary artery diameter [76].

A novel medical device has been introduced to generate an FAI-derived metric corrected for the factors mentioned above (FAI-Score). This device is a CE-marked post-processing software system that provides measures of coronary inflammation (FAI and FAI-Score) and cardiac mortality risk from CCTA DICOM images [9]. CCTA images can be sent electronically from the hospital picture archiving and communication system (PACS) through a gateway. Segmentation of epicardial and pericoronary adipose tissue is conducted using artificial intelligence (AI) [77]. For quality assurance purposes, a trained analyst checks and corrects the segmentations. The main outputs of the device are the FAI and the FAI-Score for the proximal segments of the major coronary arteries and the CaRi-Heart Risk. The CaRi-Heart Risk quantifies the risk of a fatal cardiac event at 8 years post-CCTA, including FAI-Score in a prognostic model along with clinical risk factors (smoking, diabetes, hyperlipidemia, and hypertension) and coronary plaque burden described by the modified Duke CAD index. Results are sent back to referring healthcare professionals as electronic reports [9].

FAI-Score was prognostic of cardiac mortality around each of the three major coronary vessels, in contrast to FAI, which lost its prognostic ability when measured around the LCx. Age-specific nomograms and percentile curves were generated for FAI-Score around each of the major coronary arteries as reference diagrams to individually interpret FAI-Score in clinical practice [9]. The ability of FAI-Score to detect inflammatory risk has been also assessed in the large ORFAN cohort comprising about 40,000 individuals followed up for a median 2.7-year period. In the ORFAN population, the FAI-Score in each of the three coronary vessels predicted MACE and cardiac mortality independently from CVD risk factors and CAD presence. Adding inflammatory AI risk to a model including QRISK3 and CAD-RADS 2.0 significantly improved CVD risk prognostication [78]. Notably, coronary inflammation-based CVD risk stratification was associated with higher net clinical benefit compared to stratification guided by standard prognostic models for both low- and high-risk patients [9].

**Figure 2:**
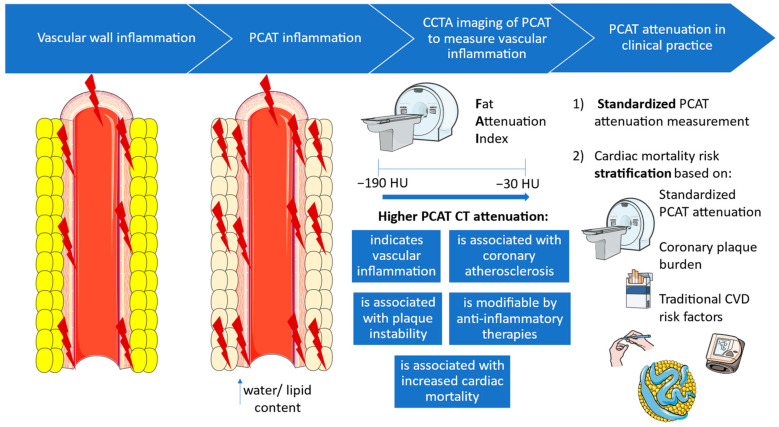
PCAT attenuation, a promising biomarker––from biology to clinical practice. CCTA: coronary computed tomography angiography, CT: computed tomography, CVD: cardiovascular disease, HU: Hounsfield units, PCAT: pericoronary adipose tissue.

Thus, this software generates a standardized metric of coronary inflammation, FAI-Score, which, in combination with coronary plaque burden and established CVD risk factors, may contribute to personalized cardiac risk stratification, superior to that provided by established prognostic models. In this way, every CCTA performed for clinical purposes could be analyzed for FAI measurement aimed at precise CVD risk stratification. Incorporation of the inflammatory burden into CVD risk prognostication would be of interest, especially in patients with increased inflammatory burden, such as patients with diabetes mellitus, autoimmune disorders, or chronic infections, as well as patients without coronary atherosclerosis, who may be reclassified to higher risk subgroups after FAI calculation [79,80,81]. Risk models developed for inflammatory risk measurement are based on cohorts undergoing clinically indicated CCTA. Consequently, to make general population screening feasible, relevant models should be created based on data from asymptomatic individuals. Whether FAI can guide the choice of patients that could be candidates for preventive treatment (either statins or anti-inflammatory agents such as colchicine) remains to be assessed by large clinical trials. The cost-effectiveness of FAI-based risk stratification in clinical practice also needs to be explored by health economics studies. Figure 2 summarizes the integration of PCAT attenuation in clinical practice as a diagnostic and prognostic biomarker of CAD.

## 9. Conclusions

Perivascular fat and the adjacent vascular wall are in a bidirectional communication, as PVAT exerts paracrine effects on the latter (outside-in) but is also influenced by inflammatory mediators generated in the vascular wall (inside-out). These mediators promote adipocyte dedifferentiation and lipolysis, and thus lead to a change in PCAT composition (increased water/lipid ratio). A fat attenuation index biomarker, measuring the mean PCAT attenuation around a defined coronary artery segment, has been developed to detect these morphological changes in computed tomography images as an increase in PCAT attenuation. The fat attenuation index has been investigated as a diagnostic and a prognostic biomarker in several studies, which indicated that it reliably flags coronary inflammation, predicts the progression of coronary plaques, enhances cardiac risk stratification, and may be modified by treatments with anti-inflammatory effects. A regulatory-cleared medical device has been developed to integrate perivascular fat inflammation into clinical practice. This device provides standardized fat attenuation index values (FAI-Score) and projects them onto age- and gender-specific nomograms for clinical evaluation. Thus, PCAT attenuation represents a promising imaging biomarker, applicable in clinical practice, that may contribute to personalized cardiovascular risk stratification and guide patient selection for targeted preventive measures to lower coronary inflammation. Large randomized clinical trials need to be conducted before PCAT attenuation guides therapeutic management.

## Figures and Tables

**Table 1:** **Types and characteristics of different adipose tissue types.** BeAT: beige adipose tissue, FABP4: Fatty Acid Binding Protein 4, PGC-1α: Peroxisome Proliferator-Activated Receptor Gamma Coactivator 1-Alpha, PPARγ: Peroxisome Proliferator-Activated Receptor Gamma, PRDM16: PR Domain Containing 16, SREBP1c: Sterol Regulatory Element-Binding Protein 1c, UCP1: Uncoupling Protein 1.

	WAT	BAT	BeAT
**Location**	SAT, abdominal VAT, thoracic VAT including EAT and PVAT	Interscapular, paravertebral, perirenal, supraclavicular	Same as WAT
**Cell shape**	Spherical	Elliptical	Spherical
**Cell morphology**	Large, single lipid droplet, contains few mitochondria	Smaller than WAT, multiple lipid droplets, contains large number of mitochondria	Small lipid droplets after stimulation, contains moderate to large number of mitochondria after stimulation
**Role**	Energy storage, thermal insulation, mechanical protection, adipokine secretion	Thermogenesis (non-shivering), anti-inflammatory effects, adipokine secretion	Thermogenesis after stimulation, adipokine secretion
**Activators**	PPARγ, SREBP1c, FABP4	UCP1, PGC-1α, PRDM16	Similar to BAT
